# R-Spondin 1/Dickkopf-1/Beta-Catenin Machinery Is Involved in Testicular Embryonic Angiogenesis

**DOI:** 10.1371/journal.pone.0124213

**Published:** 2015-04-24

**Authors:** Maria Caruso, Francesca Ferranti, Katia Corano Scheri, Gabriella Dobrowolny, Fabio Ciccarone, Paola Grammatico, Angela Catizone, Giulia Ricci

**Affiliations:** 1 Department of Anatomy, Histology, Forensic Medicine and Orthopedics—Section of Histology and Medical Embryology, Sapienza University of Rome, Rome, Italy; 2 Italian Space Agency, Rome, Italy; 3 Center for Life Nano Science@Sapienza, Istituto Italiano di Tecnologia, Rome, Italy; 4 Department of Cellular Biotechnologies and Hematology, Sapienza University of Rome, Rome, Italy; 5 Department of Molecular Medicine, Sapienza University of Rome, Rome, Italy; 6 Department of Experimental Medicine—Histology and Embryology Laboratory, Second University of Naples, Naples, Italy; University of Florida, UNITED STATES

## Abstract

Testicular vasculogenesis is one of the key processes regulating male gonad morphogenesis. The knowledge of the molecular cues underlining this phenomenon is one of today’s most challenging issues and could represent a major contribution toward a better understanding of the onset of testicular morphogenetic disorders. R-spondin 1 has been clearly established as a candidate for mammalian ovary determination. Conversely, very little information is available on the expression and role of R-spondin 1 during testicular morphogenesis. This study aims to clarify the distribution pattern of R-spondin 1 and other partners of its machinery during the entire period of testicular morphogenesis and to indicate the role of this system in testicular development. Our whole mount immunofluorescence results clearly demonstrate that R-spondin 1 is always detectable in the testicular coelomic partition, where testicular vasculature is organized, while Dickkopf-1 is never detectable in this area. Moreover, organ culture experiments of embryonic male UGRs demonstrated that Dickkopf-1 acted as an inhibitor of testis vasculature formation. Consistent with this observation, real-time PCR analyses demonstrated that DKK1 is able to slightly but significantly decrease the expression level of the endothelial marker *Pecam1*. The latter experiments allowed us to observe that DKK1 administration also perturbs the expression level of the *Pdgf-b* chain, which is consistent with some authors’ observations relating this factor with prenatal testicular patterning and angiogenesis. Interestingly, the DKK1 induced inhibition of testicular angiogenesis was rescued by the co-administration of R-spondin 1. In addition, R-spondin 1 alone was sufficient to enhance, in culture, testicular angiogenesis.

## Introduction

Vasculogenesis of the mammalian male gonad is one of the first features, besides testicular cord organization, that allows to morphologically distinguish a testis from an ovary [1–3]. In the mouse, during testicular morphogenesis, endothelial cells start to migrate from the mesonephros at the same time as cords begin to organize[1;4]. It has been demonstrated by Combes and co-authors in 2009 that endothelial cell migration and testicular cord formation are actually related and interdependent processes. In fact, at 11.5 days post coitum (dpc) mesonephric endothelial cells, due to the accumulation in the testis of chemotactic signals, start to migrate through the gonad towards the coelomic domain. Migrating mesonephric endothelial cells subdivide the gonad into approximately ten avascular regions, where testis cords form and, at the same time, define the testicular interstitial vascularised compartments. When migrating, endothelial cells reach the testicular coelomic surface, they aggregate in the coelomic domain to form the major testicular artery that runs the length of the testicular anti-mesonephric border [4;5]. Therefore, endothelial cell migration appears to be fundamental to drive the two different key processes of testicular early development: testicular cord formation and testis vascularisation. It is worth highlighting that gonad vasculogenesis time of appearance and pattern are sexually dimorphic: in particular, the embryonic ovary typically recruits vasculature by a classical angiogenic branching process, whilst the embryonic testis recruits vasculature by a disassembly of the existing mesonephric vessels [5]. Despite a growing body of data on the molecular cues regulating these specific processes, the signaling pathway cross-talk responsible for the harmonic coordination of these morphogenetic events needs to be better defined [6;7].

R-spondins (RSPOs) are a group of secreted proteins that enhance WNT/β-catenin signaling and have pleiotropic functions in development and stem cell growth. The R-spondin protein family is conserved among vertebrates and consists of four related members R-spondin1–4 (RSPO1–4) [8–13]. These proteins are able to promote β-catenin stabilization binding the LGR4, LGR5, and LGR6 receptors [14;15]. Although RSPOs are unable to initiate WNT signaling, it is known that they can potently enhance responses to low-dose WNT proteins. Dickkopf homolog 1 (DKK1) is another player in this complex machinery. This molecule is a well known inhibitor of the canonical WNT/RSPO signaling pathway. It has the ability to form a complex between LRP5/6 and Kremen1, that are RSPO1 co-receptors, and to trigger LRP5/6 internalization. This phenomenon leads to the inhibition of cell responsiveness both to WNT and RSPO cues. Significantly, it has been reported that a high concentration of RSPOs can rescue this inhibition, interfering with LRP6 internalization [16]. It is well known that WNT signaling is important in almost every fate decision during embryonic development throughout the animal kingdom [17]. Moreover, RSPOs have been shown to be dynamically and broadly expressed in a wide variety of embryonic tissues: this observation has predicted pleiotropic roles, still poorly understood, for RSPO cues during embryogenesis [13]. In short, a review of the literature clearly indicates that a balance between RSPOs and DKK1 availability provides a dynamic modulation of WNT signaling in the regulation of the harmonic control of mammalian embryonic development [18–20].

To date, both WNT4 and RSPO1 have been shown to play key roles in female gonad determination, in mice and humans, indicating that their expression and molecular role are conserved at least among mammals and that mouse models could represent a valuable research tool to provide insights into this matter [7;12;21–29]. In particular, it has been demonstrated that female *Rspo*1 knock-out mice displayed masculinized features such as genital duct pseudohermaphroditism, foetal oocyte depletion, and male specific coelomic vasculature [28] with similar characteristics to that of female *Wnt4* mutant mice [29].

Although the involvement of these two molecules in ovarian development has been extensively investigated, the function of the WNT/RSPO pathway in the development of the male gonad has been largely disregarded due to the lack of a male specific altered phenotype in the so far generated mouse models. To this purpose, we may highlight that gonad morphogenesis is a highly controlled process regulated by redundant cues: for this reason it is often difficult to determine the role of molecules involved in this process using mutant or knock-out mice. Herein we report, for the first time, a complete description, by whole mount immunofluorescence, of RSPO1/DKK1/β-catenin machinery expression pattern during the entire period of testis development. Moreover, we show a clear indication, using *ex vivo* testicular organ cultures, of a sensitive and subtle control of testicular angiogenesis exerted by a complex balance between RSPO1 and DKK1 availability occurring in the developing testis.

## Materials and Methods

### Animals

CD-1 mouse embryos were used for all experiments. Pregnant mice were housed at the DAHFMO stabulation facility of “Sapienza” University of Rome. All animal studies were conducted in accordance with the principles and procedures outlined in the National Institute of Health (NIH) Guide for Care and Use of Laboratory Animals. The protocol was approved by the Committee on the Ethics of Animal Experiments of the "Sapienza" University of Rome (legal provision art. 116/92). Mice were killed by CO_2_ asphyxiation. To determine the embryonic age, the morning after vaginal plug formation was considered as day 0.5 of embryonic development.

### Primordial Germ Cell (PGC) purification

PGCs were immunopurified from 13.5 dpc mouse embryo male gonads using the method of Pesce and De Felici [30] with some modification [31]. After extensive washing, alkaline phosphatase staining was used to estimate PGC purity, (approximately 90%). Purified PGCs were used for western blot analyses and for immunofluorescence experiments (see below).

### Western Blotting experiments

Foetal gonads were dissected by CD-1 mouse embryos at 13.5, 16.5 and 18.5 dpc. Male gonads were separated from their respective mesonephroi, and both tissues were used for western blot analysis. Foetal tongues were also dissected by 13.5 dpc CD-1 mouse embryos and PGCs were obtained from male embryos of the same developmental age (see above). 17.5 dpc intestine and SKOV3 cells were used as western blot negative controls of RSPO1 [13] and DKK1 [32] respectively. All the organs and cells were homogenised in TRIS/HCl pH 7.4/1% SDS, and protease inhibitors (P8340, Sigma-Aldrich, St. Louis, Missouri, USA) were added. Protein contents were determined using the Bradford protein assay (Biorad Laboratories, Hercules, California, USA). Proteins (70 μg per lane) were re-suspended in boiling Laemmli buffer under reducing conditions, separated on 10% SDS–PAGE gel, and then electrotransferred to nitrocellulose membrane (Protran, Dassel, Germany, Europe). Non-specific antibody binding was blocked by incubation with 5% no-fat milk in TBS buffer (20 mM Tris pH 7.6, 150 mM NaCl) for 16 hours at 4°C. After quenching, membranes were incubated with anti-RSPO1 (1:1000 dilution, goat polyclonal, AF3474, R&D System, Minneapolis, Minnesota, USA), anti-DKK1 (1:200 dilution, goat polyclonal, sc-30785, Santa Cruz Biotechnology, Heidelberg, Germany, Europe), or anti-α-tubulin (1:1000 dilution, mouse monoclonal, T5168, Sigma-Aldrich, St. Louis, Missouri, USA). The membranes were then incubated with the appropriate AP-conjugated secondary antibody (rabbit anti-goat, 1:3000 dilution, A4187, Sigma-Aldrich or rabbit anti-mouse, 1:3000 dilution, A4312, Sigma-Aldrich, St. Louis, Missouri, USA) for 1 hour at room temperature (RT). Immunocomplexes were detected using a Western blot chemiluminescent reagent (CDP-star, Perkin Elmer, Waltham, Massachusetts, USA) following the manufacturer’s instructions.

### Organ culture experiments

Urogenital ridges (UGRs), isolated from 12.5 dpc male embryos, were cultured for 3, 6 or 48 hours on steel grids previously coated with 2% agar and placed in organ culture dishes (Falcon Bergen County, New Jersey, USA). Dulbecco’s modified Eagle’s medium (DMEM, Sigma-Aldrich, St. Louis, Missouri, USA) supplemented with glutamine (2mM, Gibco, Monza, Italy; Europe), penicillin-streptomycin (100 IU/ml-100 mg/ml, Gibco, Monza, Italy; Europe) was used. Recombinant Mouse RSPO1 (3474-RS-050, R&D Systems, Minneapolis, Minnesota, USA) at a concentration of 1,5 or 3 μg/ml, or Recombinant Mouse DKK1 (5897-DK-010/CF, R&D Systems, Minneapolis, Minnesota, USA) at a concentration of 1, 2, 4 μg/ml, was added to the culture medium of treated UGRs, while the contra-lateral organs were cultured in medium alone. Foetal male UGRs were cultured at 37°C in a humidified atmosphere of 5% CO_2_. At the end of the culture time, samples were then fixed overnight by immersion in 4% paraformaldehyde (PFA) in Phosphate Buffered Saline (PBS) (pH 7.4) and then processed for immunofluorescence experiments (see below). Alternatively, cultured samples were used for quantitative RT Polymerase Chain Reaction assay.

### Whole mount immunofluorescence

Male UGRs dissected by CD-1 mouse embryos at different developmental stages (from 11.5 to 18.5 dpc), or 12.5 dpc cultured male UGRs (see above), were processed by whole mount immunofluorescence experiments. Also foetal tongues (13.5 dpc) and female UGRs (15.5 dpc) were dissected and processed for whole mount immunofluoresce experiments. All samples were fixed by overnight immersion in 4% PFA in PBS (pH 7.4) at 4°C, permeabilized in PBS/1% BSA/0.1% Triton for 1 hour, and blocked first in 1 M Glycine (pH 8) for 1 hour and then in PBS/1% BSA/5% donkey serum for 4 hours. Samples were then incubated overnight with the following primary antibodies: anti-RSPO1 (1:20, goat polyclonal, AF3474, R&D Systems, Minneapolis, Minnesota, USA), anti-DKK1 (1:100, rabbit polyclonal, ab61034, abcam, Cambridge, UK, Europe; 1:50, goat polyclonal, sc-30785, Santa Cruz Biotechnology, Heidelberg, Germany, Europe), anti-β-catenin (1:50, mouse monoclonal, sc-7963, Santa Cruz Biotechnology, Heidelberg, Germany, Europe), anti-LGR4 (1:50, rabbit polyclonal, sc-292344, Santa Cruz Biotechnology, Heidelberg, Germany, Europe), anti-Kremen1 (1:50, rabbit polyclonal, sc-99034, Santa Cruz Biotechnology, Heidelberg, Germany, Europe), anti-CD31/PECAM1 (1:250, rat monoclonal, 550274, BD Pharmingen, Bergen County, New Jersey, USA), anti-VASA (1:200, rabbit polyclonal, ab13840, abcam, Cambridge, United Kingdom, Europe), anti-Ki67 (1:100, rabbit polyclonal, ab15580, abcam, Cambridge, United Kingdom, Europe), anti-cleaved Caspase-3 (1:200, rabbit polyclonal, #9661, Cell Signaling, Danvers, Massachusetts, USA), anti-p-Histone H3 (1:50, mouse monoclonal, sc-374669, Santa Cruz Biotechnology, Heidelberg, Germany, Europe); all the primary antibodies were diluted in PBS/1% BSA/0.1% Triton. Samples were then washed three times with PBS/1% BSA/0.1% Triton and incubated for 1 hour and 30 minutes with the appropriate secondary antibodies (Jackson ImmunoResearch Lab, West Grove, Pennsylvania, USA): FITC–conjugated donkey anti-goat IgG (705-095-147), for RSPO1 and DKK1 detection; or FITC–conjugated donkey anti-rabbit IgG (711-095-152), for DKK1, LGR4 and Kremen1 detection; or TRITC–conjugated donkey anti-mouse IgG (715-025-150), for β-catenin and Ki67 detection; or FITC–conjugated donkey anti-rat IgG (712-095-153) or Cy 5–conjugated donkey anti-rat IgG (712-175-153), for PECAM1 detection; or TRITC–conjugated donkey anti-rabbit IgG (711-025-152), for cleaved Caspase-3 and VASA detection; or FITC–conjugated donkey anti-mouse IgG (715-095-150), for p-Histone H3 detection. All the secondary antibodies were diluted 1:200 in PBS, except for the Cy 5–conjugated donkey anti-rat IgG that was diluted 1:400. Where indicated, the samples were labelled with TO-PRO3 iodide nuclear fluorescent dye 642/661 (1:5000 in PBS, T3605, Invitrogen, Monza, Italy; Europe). As negative control, the primary antibody was omitted (see S1 Fig). All treatments were performed under continuous rotation. All the samples were mounted in buffered glycerol (pH 9.5).

### Confocal microscopy and image analysis

Leica confocal microscope (Laser Scanning TCS SP2), equipped with Ar/ArKr and HeNe lasers, was used for RSPO1, VASA, DKK1, β-catenin, Kremen1, LGR4, PECAM1, Ki67, cleaved Caspase-3 and p-Histone H3 immunolocalization. Laser lines were at 488 nm and 543 nm for FITC and TRITC respectively, and 633 nm for Cy5 and TO-PRO3 iodide excitation. The images were scanned under a 10X, 20X objective or 40X oil immersion objective. Color channels were merged and co-localizations were analysed with the Leica confocal software. To analyse the whole gonads, each sample was analysed using a spatial series through the Z axis. Each spatial series was composed of approximately 15–20 optical sections with a step size of 5–7 μm and was analysed using the Leica confocal software.

### Reverse Transcription and Relative-Quantitative RT Polymerase Chain Reaction

Total RNA was extracted (RNeasy Plus micro Kit, 74034, Qiagen, Milan, Italy) from UGRs, withdrawn from 12.5 dpc male embryos, cultured for 48 hours in medium alone or supplemented with 2μg/ml DKK1. Total RNA (1μg) was reverse-transcribed using the QuantiTect Reverse Transcription Kit (Qiagen, Milan, Italy). Relative-Quantitative RT-PCR was performed using the ABI PRISM 7500 SDS (Life Technologies—Applied Biosystems, USA), the TaqMan Universal PCR Master Mix and TaqMan Gene Expression Assay (Life Technologies, USA). Quantitative sample value was normalized for the expression of β-actin mRNA. The relative level for each gene was calculated using the 2-ΔΔCt method [33] and reported as fold change.

### Statistical analysis

Proliferation and apoptosis assay data were expressed as the mean ± standard error (SE) of at least three separate experiments. Statistical analysis was performed by Student’s t-test. Differences were considered significant at P-value <0.05.

Real time PCR statistical analysis was performed with GraphPad Prism v 5.0 software; groups were compared by Unpaired t-test and the difference between the two groups was considered significant for P value <0.05.

## Results

### RSPO1 availability during testicular morphogenesis

**Fig 1:**
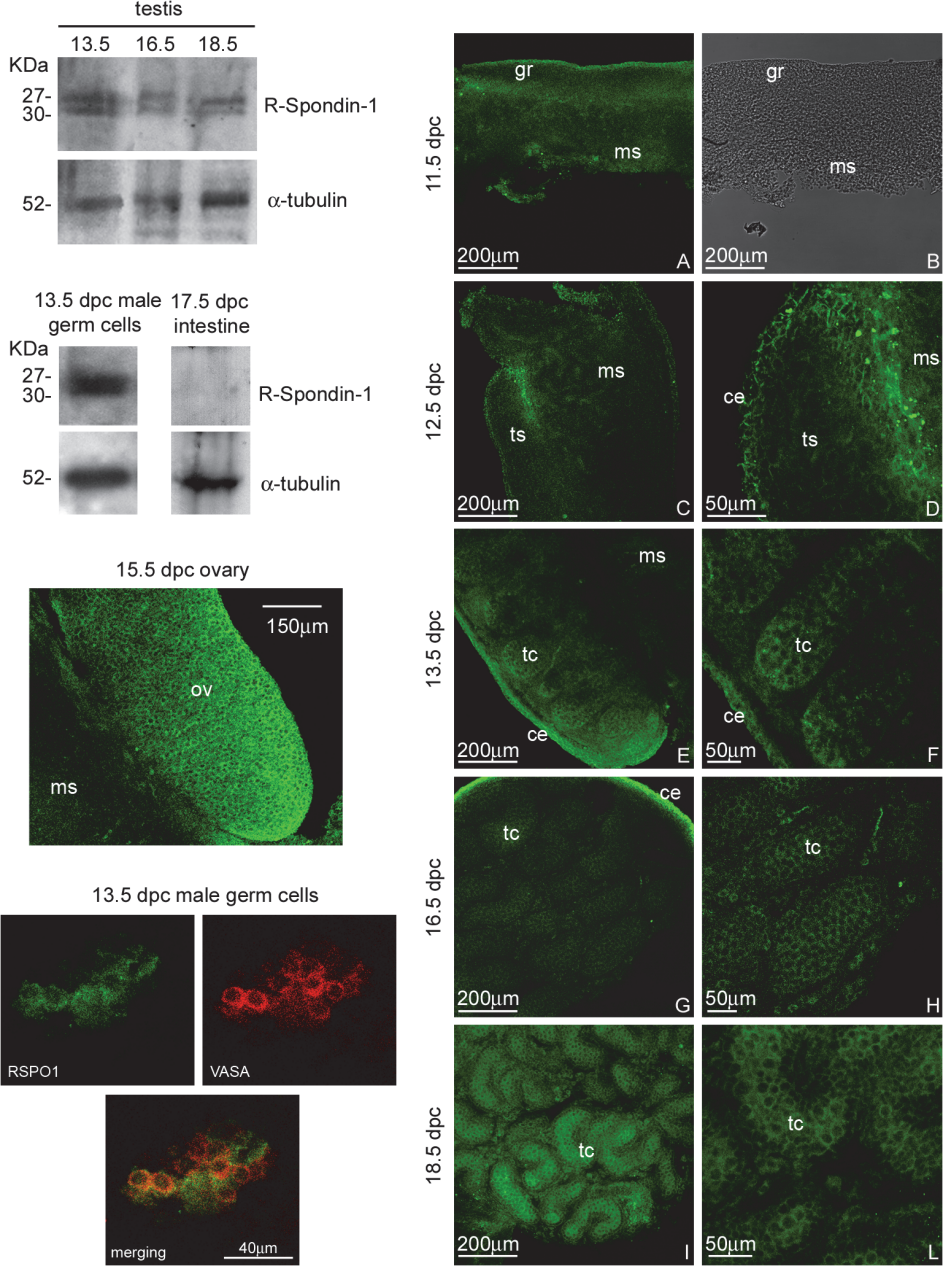
RSPO1 protein content and distribution pattern during male gonad development. Left panel: (Upper part) Western blot analysis of RSPO1 in 13.5, 16.5 and 18.5 dpc embryonic male gonads, and 13.5 dpc male germ cells. As RSPO1 western blot negative control 17.5 dpc intestine is also reported. Two different bands, corresponding to the immature (27 KDa band) and the glycosilated isoform (30 KDa band), were detected. (Lower part) Confocal microscopy analysis of RSPO1 distribution observed by whole mount immunofluorescence (FITC signal) in 15.5 dpc ovary (positive control) and in 13.5 dpc male germ cells. RSPO1 immuno-labeled germ cells were double stained with anti-VASA (germ cell marker) antibody. Right panel: Confocal microscopy analysis of RSPO1 distribution observed by whole mount immunofluorescence (FITC signal) in 11.5 (A), 12.5 (C and D), 13.5 (E and F), 16.5 (G and H), and 18.5 dpc (I and L) male UGRs at different magnifications. In B the corresponding bright field of 11.5 dpc male genital ridge is reported. ce: coelomic epithelium; gr: genital ridge; ms: mesonephros; tc: testicular cords; ts: testis, ov: ovary.

Western blot analysis on testes isolated from 13.5, 16.5 and 18.5 dpc male embryos showed that RSPO1 protein is clearly detectable in the male gonads at all the pre-natal ages considered. We detected both intracellular isoform of RSPO1, as a 27 KDa protein, as well as the glycosilated mature isoform characterized by a higher molecular weight (30KDa). PGCs, isolated at 13.5 dpc from male embryos, also express RSPO1 indicating that this cell type is responsible, at least in part, of the observed RSPO1 expression in the foetal testis. Since Nam and coworkers in 2007 [13] demonstrated that RSPO1 in not expressed during the mouse intestine development, as western blot negative control, we used 17.5 dpc foetal intestine lysates. As expected this sample does not produce a detectable level of this signal molecule (Fig 1 upper part of the left panel).

### RSPO1 distribution pattern during testicular embryonic development

UGRs isolated from 11.5, 12.5, 13.5, 16.5 and 18.5 dpc male embryos were fixed and processed for RSPO1 localization by whole mount immunofluorescence and analysed by confocal microscopy. As positive control of RSPO1 antibody, the immunofluorescence experiments were also carried out on foetal female UGRs (Fig 1 lower part of the left panel). Each experiment was performed at least in triplicate. In order to evaluate spatial distribution of RSPO1 in the entire developing organs, we performed optical spatial series at different magnifications and a total of 450 optical sections were observed. This analysis showed that at 11.5 dpc, RSPO1 appears barely detectable on the coelomic surface of the genital ridge. This signal becomes stronger and better defined at 12.5 dpc and, in addition, RSPO1 is highly expressed in the cranial part of the genital ridge-mesonephros interface. From 13.5 to 18.5 dpc, the RSPO1 signal appears mainly localized on the coelomic portion of the testis and inside the testicular cords. A representative optical section of each analysed age is shown (Fig 1 right panel). It interesting to note that male PGCs, isolated at 13.5 dpc, are clearly positive for RSPO1 even when subjected to immunofluorescence analysis. This result confirms western blot analysis results and demonstrates that male germ cells are responsible, at least in part, for the observed RSPO1 tubular staining.

### DKK1 distribution pattern during testicular embryonic development

**Fig 2:**
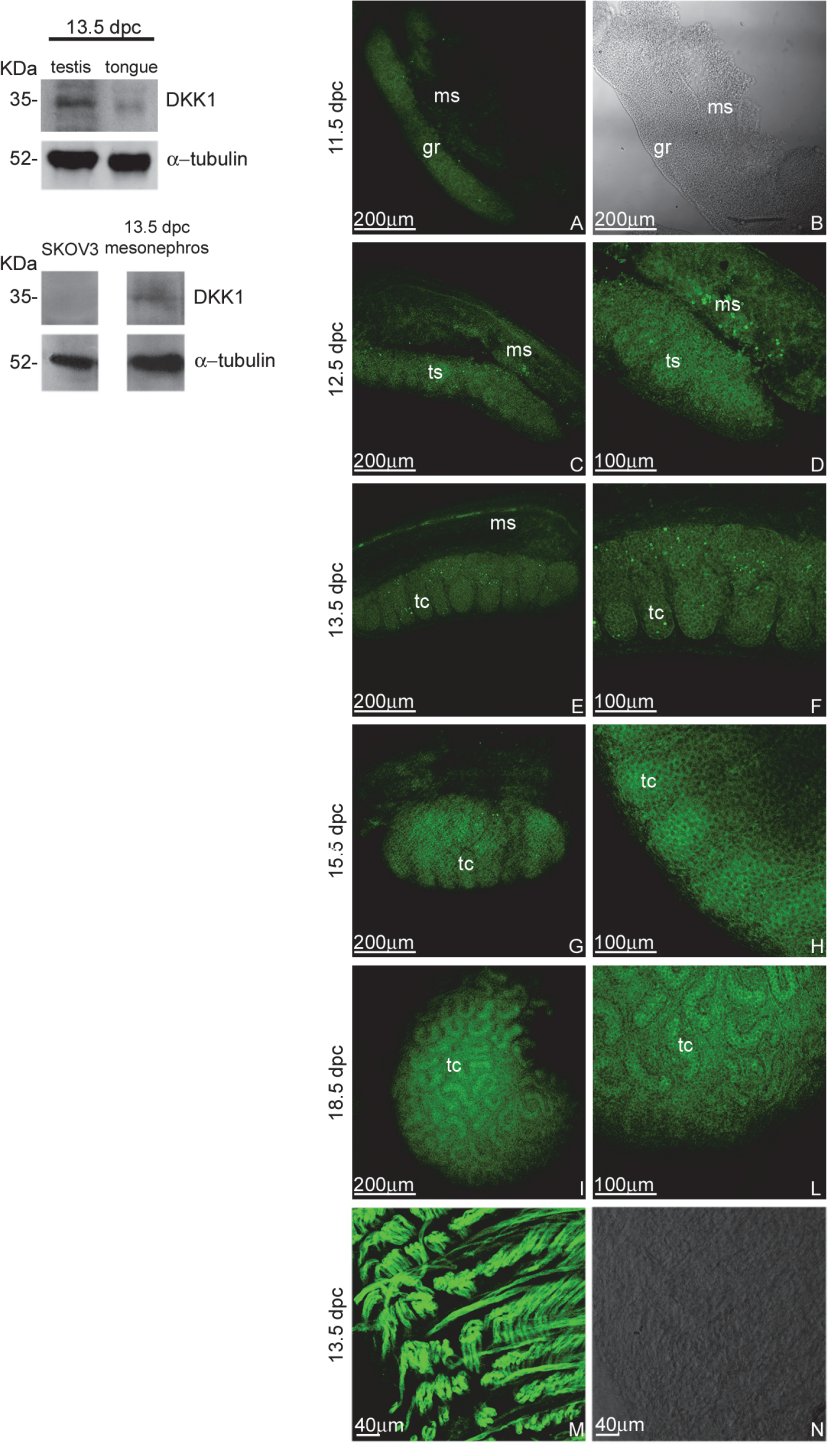
DKK1 protein content and distribution pattern during male gonad development. Left panel: Western blot analysis of DKK1 in 13.5 dpc embryonic male gonad, mesonephros, and tongue. As DKK1 western blot negative control SKOV3 cell lysates are also reported. One band, at 35 KDa molecular weight was detected. Right panel: Confocal microscopy analysis of DKK1 distribution, observed by whole mount immunofluorescence (FITC signal) in 11.5 (A), 12.5 (C and D), 13.5 (E and F), 15.5 (G and H), and 18.5 dpc (I and L) male UGRs at different magnifications. In B the corresponding bright field of 11.5 dpc male genital ridge is reported. In M the DKK1 signal in the tongue of 13.5 dpc embryos is reported as positive control. In N the corresponding bright field has been shown. gr: genital ridge; ms: mesonephros; tc: testicular cords; ts: testis.

UGRs isolated from 11.5, 12.5, 13.5, 15.5 and 18.5 dpc embryos were fixed and processed for DKK1 localization by whole mount immunofluorescence using two different antibodies (see materials and methods) (Fig 2 right panel). As positive control of DKK1 antibody, the immunofluorescence experiments were carried out also on foetal tongues (Fig 2M). Moreover, to confirm our whole mount immunofluoresce results, western blot analyses on testes, mesonephroi, and tongues of 13.5 dpc embryos were performed. As western blot negative control, SKOV3 cell lysates were used [32] (Fig 2 left panel). Spatial distribution of DKK1 in the entire developing organs was analysed by confocal microscopy. Each experiment was performed at least in triplicate. We performed optical spatial series at different magnifications and a total of 390 optical sections were observed at all analysed ages. We observed that at 11.5 dpc DKK1 appears as a diffuse signal, detectable inside the genital ridge. From 12.5 to 18.5 dpc, DKK1 is restricted to testicular cords. This localization partially overlaps RSPO1 distribution pattern. From 13.5 dpc, DKK1 is clearly detectable also as a cytoplasmic signal in few dispersed cells, mainly localized in the testicular cords. DKK1 is never detectable on the coelomic surface of the developing testes: this represents the main difference between DKK1 and RSPO1 spatial distribution. A representative optical section of each analysed stage is shown (Fig 2). This result is not apparently consistent with the previously reported *Dkk1* expression data performed by in situ hybridization [34]. These authors found *Dkk1* mRNA was restricted to the coelomic portion of the testis. These results were difficult to combine with our observations which aimed to assess the availability of DKK1 protein during testicular embryonic development. To ensure consistency and soundness to our data, we used two different antibodies obtaining the same results. We hypothesize that, during testis development, the *Dkk1* mRNA, observed by Combes et coll. in 2011 on the coelomic surface, is actually negatively regulated and/or not translated. It is well known, in fact, that the presence of mRNA does not necessarily denote correct translation of the protein. Furthermore, DKK1 is a secreted protein and the obtained results may suggest efficient diffusion from the embryonic region where it is synthesized, to the areas where the DKK1 receptors are accessible.

### β-catenin distribution pattern during testicular embryonic development

**Fig 3:**
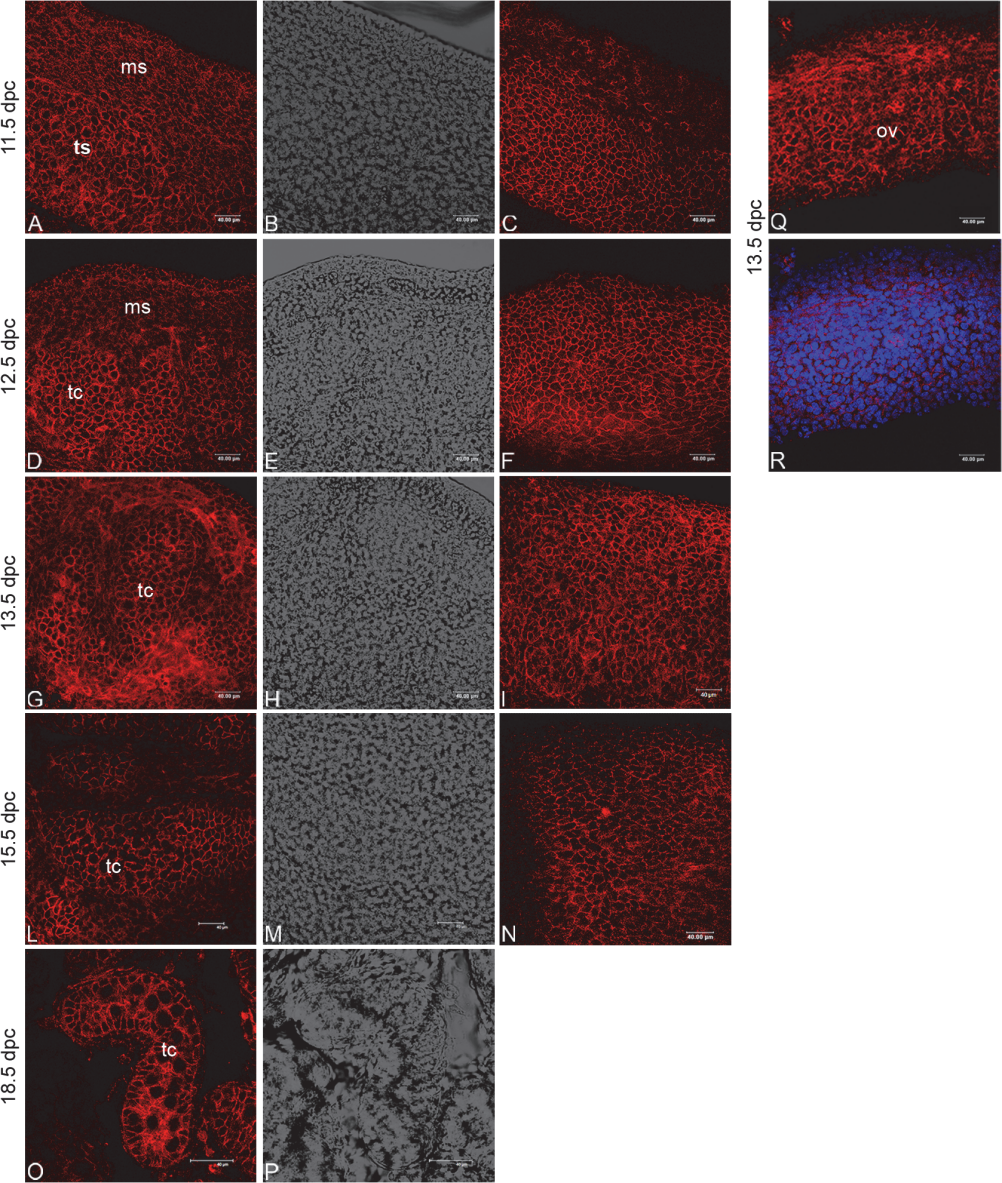
β-catenin distribution pattern during male gonad development. Confocal microscopy analysis of β-catenin distribution, observed by whole mount immunofluorescence in 11.5, 12.5, 13.5, 15.5 and 18.5 dpc male urogenital ridges. TRICT signal indicates β-catenin staining in the different prenatal ages: C, F, I, and N show representative optical sections withdrawn at the organ surface while A, D, G, L, and O show representative optical sections withdrawn in the inner part of the same organs. In B, E, H, M, and P the corresponding bright fields are also reported. As positive control the β-catenin distribution pattern in 13.5 dpc ovary (Q) is shown. In R the same image merged with nuclei staining is also reported. ms: mesonephros; tc: testicular cords; ts: testis; ov: ovary.

UGRs isolated from 11.5, 12.5, 13.5, 15.5 and 18.5 dpc embryos were fixed and processed by whole mount immunofluorescence to detect β-catenin spatial distribution. Each experiment was performed at least in triplicate. To image the full thickness of the organs optical spatial series, using a 40X objective, were performed and a total of 440 optical sections were observed at all analysed ages. At 11.5 dpc β-catenin appears as a cortical cytoplasmic signal on the coelomic epithelium. From 12.5 to 18.5 dpc, β-catenin is detectable both on the coelomic epithelium region and inside the testicular cords as a cortical cytoplasmic signal on the epithelial cells. The pattern observed in the cords strongly suggest that both Sertoli and germ cells express β-catenin in all the developmental ages analysed. A representative optical section of each analysed stage is shown (Fig 3). The antibody used was able to recognize both the cytoplasmic and nuclear isoform of β-catenin, however, β-catenin in the nucleus of testicular cells was never observed in the freshly dissected developing testes analysed (Fig 3A–3P). To better understand the implications of this observation, we used the same antibody to detect this protein on developing ovaries, as it is well known that the canonical Wnt/β-catenin signaling pathway is clearly implicated in ovarian development. Surprisingly, β-catenin nuclear staining is occasionally also observed in the female gonad (Fig 3Q and 3R). Combining these observations we suggest that β-catenin may translocate very rapidly, *in vivo*, and, for this reason, could be difficult to report. An alternative hypothesis is that, in the testis, the RSPO1 signaling pathway activation is not necessarily mediated by β-catenin nuclear translocation in all the target cells. By following this hypothesis, some target cells may use RSPO1-activated β-catenin as a cytoplasmic adaptor protein.

### Kremen1 and LGR4 distribution pattern on 12.5 and 13.5 dpc testes

UGRs isolated from 12.5 and 13.5 dpc embryos were fixed, processed using whole mount immunofluorescence and analysed by confocal microscopy for Kremen1 and LGR4 detection.

**Fig 4:**
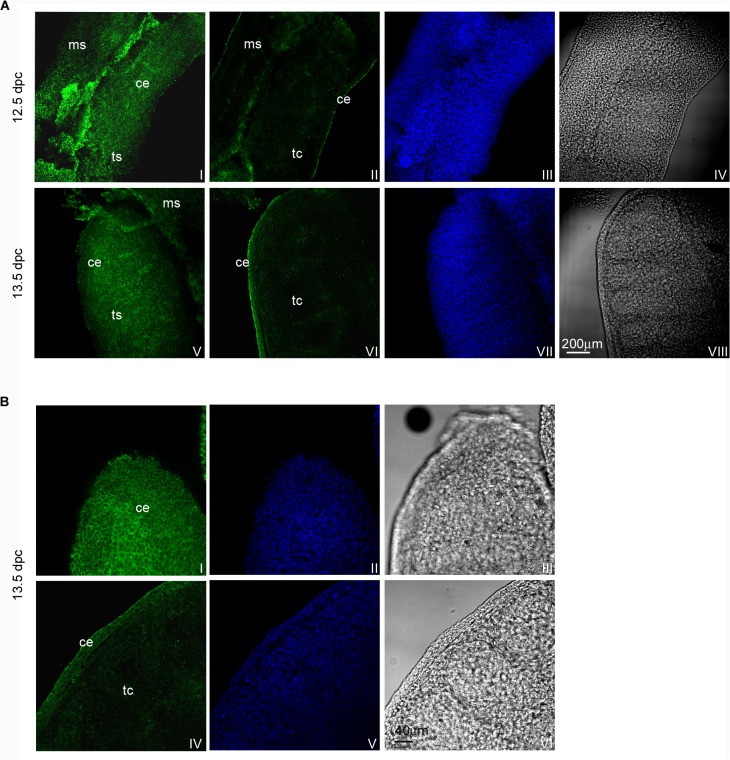
Kremen1 distribution pattern in 12.5 and 13.5 dpc male gonads. In panel A confocal microscopy analysis of Kremen1 distribution pattern, observed by whole mount immunofluorescence in 12.5 and 13.5 dpc male UGRs is reported. Kremen1 signal (FITC) detectable on the celomic surface of 12.5 and 13.5 dpc is shown in (I) and (V) respectively; the signal detectable in the inner part of 12.5 and 13.5 dpc male gonad is reported in (II) and (VI); the corresponding TO-PRO3 staining (nuclei) is reported in (III) and (VII) and the corresponding bright fields are shown in (IV) and (VIII). In panel B a higher magnification of 13.5 dpc testis Kremen1 immunofluorescence is reported. The signal detectable in the coelomic portion of the gonad is shown in (I) whereas the signal in the inner part of the testis is shown in (IV); the corresponding TO-PRO3 staining (nuclei) is reported in (II) and (V) and the corresponding bright fields are shown in (III) and (VI). ce: celomic epithelium; ms: mesonephros; tc: testicular cords; ts: testis.

**Fig 5:**
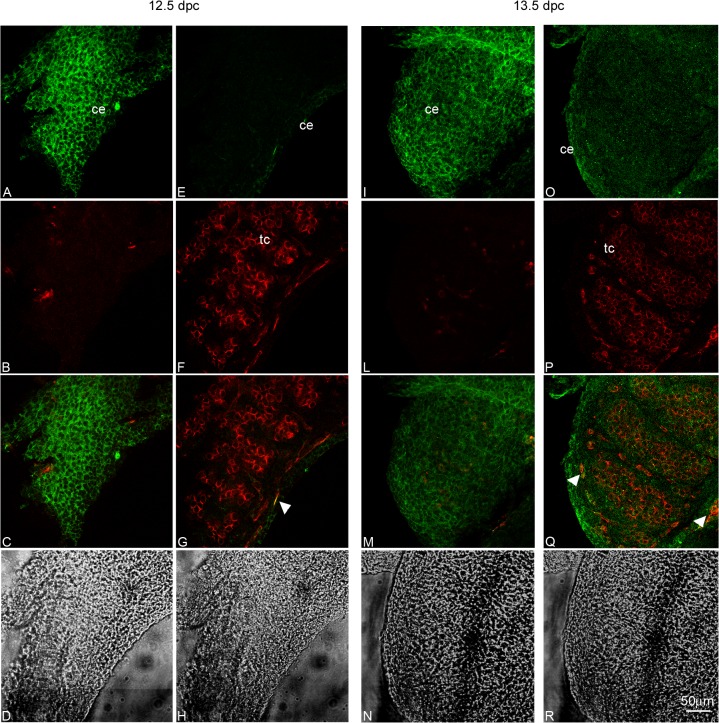
LGR4 distribution pattern in 12.5 and 13.5 dpc male gonads. Confocal microscopy analysis of LGR4 localization, observed by whole mount immunofluorescence in 12.5 and 13.5 dpc male UGRs. In the left part of the panel LGR4 signal (FITC) and PECAM1 signal (Cy5) on the coelomic surface (A and B) and in the inner part (E and F) of 12.5 dpc male gonad are reported; images C and G show the merging images of FITC and Cy5 signals while D and H show the corresponding bright fields. In the right part of the panel, LGR4 signal (FITC) and PECAM1 signal (Cy5) on the coelomic portion (I and L) and in the inner part (O and P) of 13.5 dpc male gonad are reported; images M and Q show the merging images of FITC and Cy5 signals while N and R show the corresponding bright fields. White arrowheads indicate LGR4 positive endothelial cells. ce: coelomic epithelium; tc: testicular cords.

Optical spatial series were performed and a total of 224 or 150 optical sections were analysed for Kremen1 and LGR4 immunodetection respectively. We observed that Kremen1 was restricted to the coelomic surface of both 12.5 dpc and 13.5 dpc male gonads. Moreover, gonad-mesonephros interface appeared clearly positive for Kremen1, mainly at 12.5 dpc (Fig 4). LGR4 distribution is clearly detectable on the coelomic surface of the organs both in 12.5 dpc and in 13.5 dpc male gonads (Fig 5).

To determine whether the endothelial cells express LGR4 receptor, we performed double immunofluorescence experiments, using anti-LGR4 and anti-PECAM1 (endothelial/germ cell marker) antibodies. Confocal microscopy analysis showed that some endothelial cells express LGR4 (Fig 5, arrow heads), while germ cells are never positive for this receptor (Fig 5).

### DKK1 impairs endothelial cell branching during testicular angiogenesis

To study the endothelial cell distribution pattern during different stages of testicular development, we performed whole mount immunofluorescence experiments analysing, by confocal microscopy, the endothelial cell marker PECAM1. These experiments were performed on UGRs isolated from 12.5, 13.5 and 15.5 dpc male embryos. In testes isolated from 12.5 dpc embryos, the coelomic vessel is identifiable whereas only few endothelial cells are observable among the developing testicular cords (S2A and S2C Fig). In testes isolated from 13.5 dpc embryos, we observed an increase in the endothelial cell branches among the testicular cords that join the microvascular network at the coelomic domain (S2E Fig). Testes isolated from 15.5 dpc embryos appear completely vascularised (S2G Fig).

**Fig 6:**
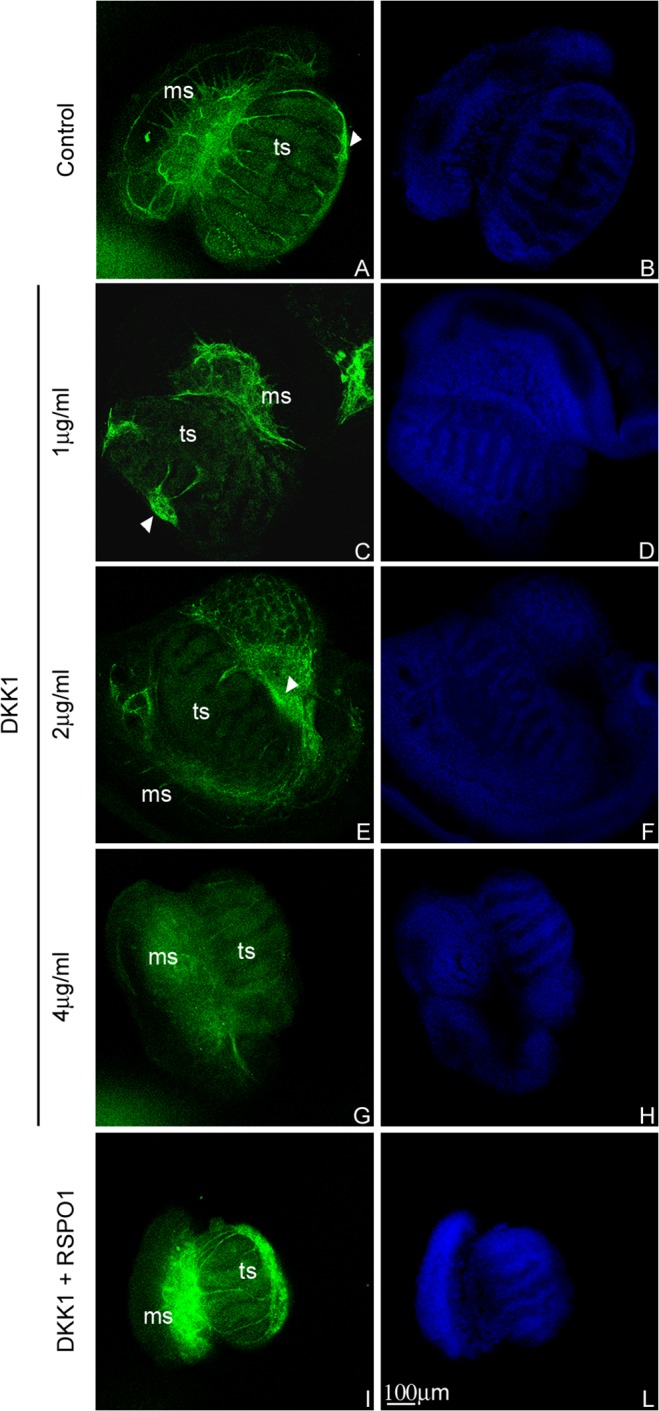
PECAM1 distribution pattern in cultured 12.5 dpc male UGRs. Confocal microscopy analysis of PECAM1 localization, observed by whole mount immunofluorescence in 12.5 dpc male gonads cultured for 48 hours in medium alone (A), in the presence of DKK1 at concentration of 1 μg/ml (C), 2 μg/ml (E) and 4 μg/ml (G) and in the presence of DKK1/RSPO1 at concentration of 2 and 3 μg/ml respectively (I). The corresponding TO-PRO3 staining (nuclei) are reported in B,D,F,H, and L. White arrowheads indicate the coelomic vessel. ms: mesonephros; ts: testis.

To address the putative function of RSPO1/DKK1 machinery on endothelial cell branching, we performed *ex vivo* organ cultures of 12.5 dpc male UGRs. Under these experimental conditions it is possible to follow the physiological endothelial cell migration from the mesonephric anlage to the testicular domain [4]. The samples were cultured for 48 hours in medium alone or in medium added with different concentrations of DKK1 (1-2-4 μg/ml). After culture, the UGRs were fixed and processed using whole mount immunofluorescence to detect PECAM1 positive endothelial cell distribution. By confocal microscopy we performed 540 optical sections for control samples and 75, 198, 184 for DKK1 treated samples at concentrations of 1-2-4 μg/ml respectively. As expected, the control testes showed both a well-defined coelomic vessel as well as internal branched endothelial cells between testicular cords, joining the coelomic domain to the gonad-mesonephros border (Fig 6A). DKK1 treated testes (1–2 μg/ml) showed an altered microvascular network at the coelomic domain, and a reduced internal vascular branching (Fig 6C and 6E). Moreover, in 4 μg/ml DKK1 treated testes, both internal angiogenesis and coelomic vasculature organization were clearly impaired (Fig 6G).

### RSPO1 rescues DKK1 induced vascular alterations on cultured male UGRs

Since DKK1 pharmacological administration to 12.5 dpc male UGRs was able to impair testis angiogenesis, we wondered whether RSPO1 might act as an antagonist of this effect. Therefore, we performed the same organ culture experiments analysing an additional culture condition in which we compared the effect of DKK1 administered alone with the co-administration of DKK1 (2μg/ml) and RSPO1 (3μg/ml). As previously described, by confocal microscopy analyses, we observed a reduced internal vascular branching in DKK1 treated testes; interestingly, in the contra-lateral samples, in which we had administered both DKK1 and RSPO1, we observed a larger number of internal migrated endothelial cells, comparable in patterning to the control samples cultured in medium alone (Fig 6I).

### DKK1 does not influence testicular cell proliferation

To determine whether DKK1, administered to culture medium, may influence cellular proliferation, we performed immunofluorescence experiments for phosphoHistoneH3 (M-phase marker) and Ki67 (proliferation marker) in 12.5 dpc male UGRs cultured for 48 hours in the presence or absence of DKK1 at concentrations of 2 and 4μg/ml respectively.

**Fig 7:**
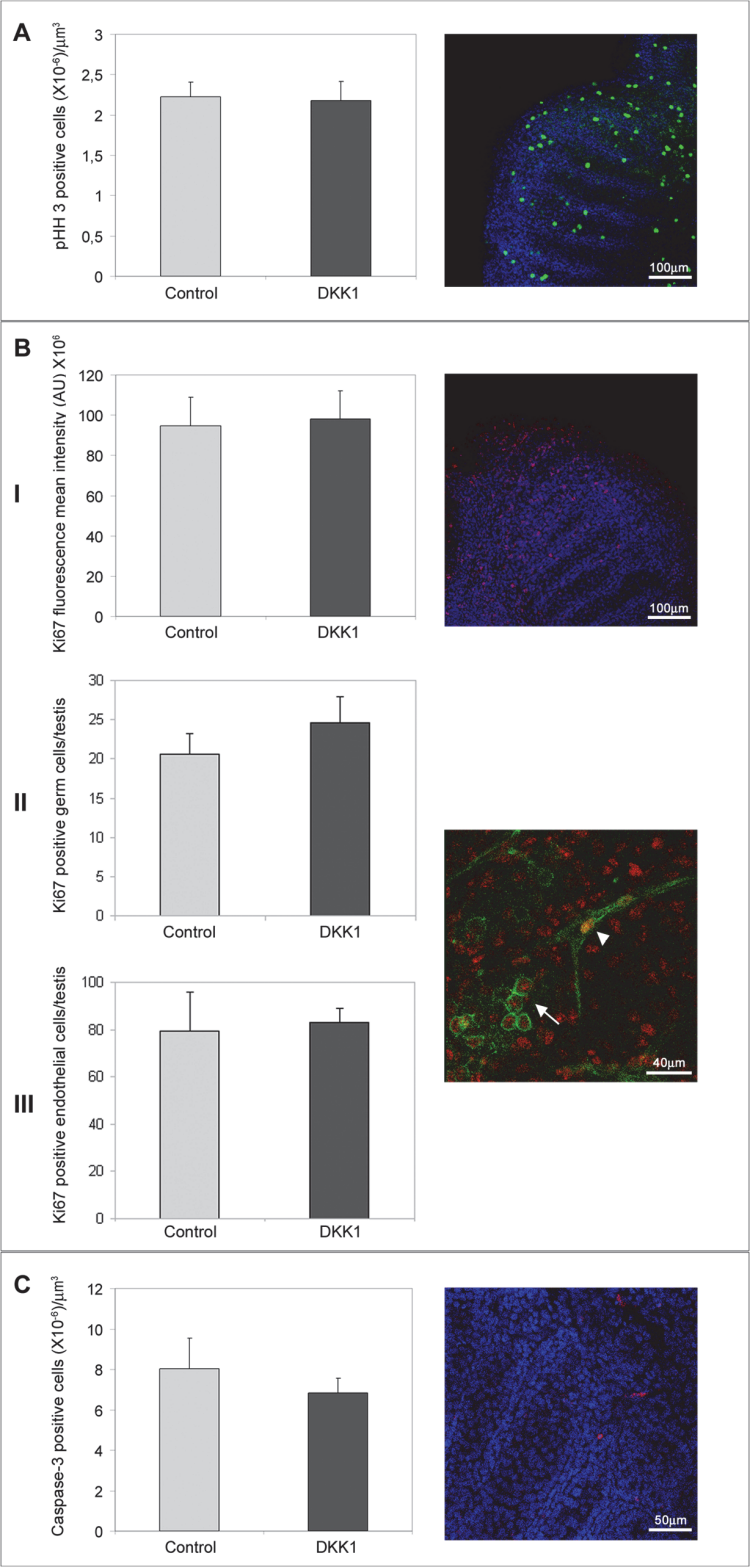
Proliferation and apoptosis indexes in cultured 12.5 dpc male gonads. In the panel A, a graph indicating the number of pHistone H3 positive cells/μm^3^ observed in testes cultured for 48 hours in medium alone (control) and in presence of 2 μg/ml DKK1 is reported. Statistical analysis, evaluated by Student's T test, was not significant. A representative merging optical section of pHistone H3 signal (FITC) and TO-PRO3 staining (nuclei) is reported in the right part of the panel. In the panel B, a graph indicating the quantitative analysis of Ki67 fluorescence intensity observed in testes cultured for 48 hours in medium alone (control) and in presence of 4 μg/ml DKK1 is reported in (I). Statistical analysis, evaluated by Student's T test, was not significant. A representative merging optical section of Ki67 signal (TRITC) and TO-PRO3 staining (nuclei) is reported in the right part of the panel. In (II) a graph indicating the number of Ki67 positive germ cells per testis is reported (Ki67/PECAM1 double positive cells inside the testicular cords). In (III) a graph indicating the number of Ki67 positive endothelial cells per testis is reported (Ki67/PECAM1 double positive cells in the interstitial compartment). A representative merging optical section of Ki67 signal (TRITC) and PECAM1 staining (FITC) is reported in the right part of the panels II e III. The white arrowhead indicates endothelial cells and the white arrow indicates germ cells. In the panel C the graph indicates the number of Caspase-3 positive cells/μm^3^ observed in testes cultured for 48 hours in medium alone (control) and in presence of 4 μg/ml DKK1. Statistical analysis, evaluated by Student's T test, was not significant. In the right part of the panel a representative optical section merging Caspase-3 (TRITC) and TO-PRO3 (nuclei) staining is reported.

Untreated and contra-lateral DKK1 (2 μg/ml) treated samples were fixed in 4% PFA and processed for phosphoHistoneH3 immunolocalization. By confocal microscopy analyses, we counted proliferating cells using Leica confocal software. No significant differences were observed in phosphoHistoneH3-positive cell number between the control and the DKK1treated testes (Fig 7A).

Moreover, we performed organ culture experiments in the same conditions and we processed cultured testes for Ki67 detection. By confocal microscopy we performed quantitative analyses using Leica confocal software to determine the mean fluorescence intensity of Ki67 in equivalent-sized regions of both the control and the treated samples. No significant differences were observed in 4 μg/ml DKK1 treated testes compared to the control samples (Fig 7B I). To better define the consequence of exogenous DKK1 administration on testicular germ cell and endothelial cell proliferation, we performed double immunofluorescence experiments for Ki67 and PECAM1 markers on the same samples. Then, we counted separately the Ki67/PECAM1 double positive cells detectable in the tubular (germ cells) (Fig 7B II) and in the interstitial (endothelial cells) (Fig 7B III) compartment of cultured testes. Even in this case, no significant differences in the number of proliferating cells were observed. We can conclude that DKK1 does not mediate the observed testicular vascular branching impairment by affecting endothelial cell proliferation.

### DKK1 does not influence testicular cell death

To verify whether DKK1, administered to culture medium, may influence cell death, we performed immunofluorescence experiments to detect cleaved Caspase-3. We cultured 12.5 dpc male UGRs for 48 hours in the presence or absence of the highest DKK1 concentration (4μg/ml). Untreated and contra-lateral DKK1 treated samples were fixed in 4% PFA and processed for cleaved Caspase-3 immunolocalization. By confocal microscopy analyses, we counted dead cells using Leica confocal software. No significant differences in the number of Caspase-3 positive cells were observed between the control and the DKK1 treated testes (Fig 7C).

### DKK1 influences *Pecam*1 and *Pdgf-b* expression level

**Fig 8:**
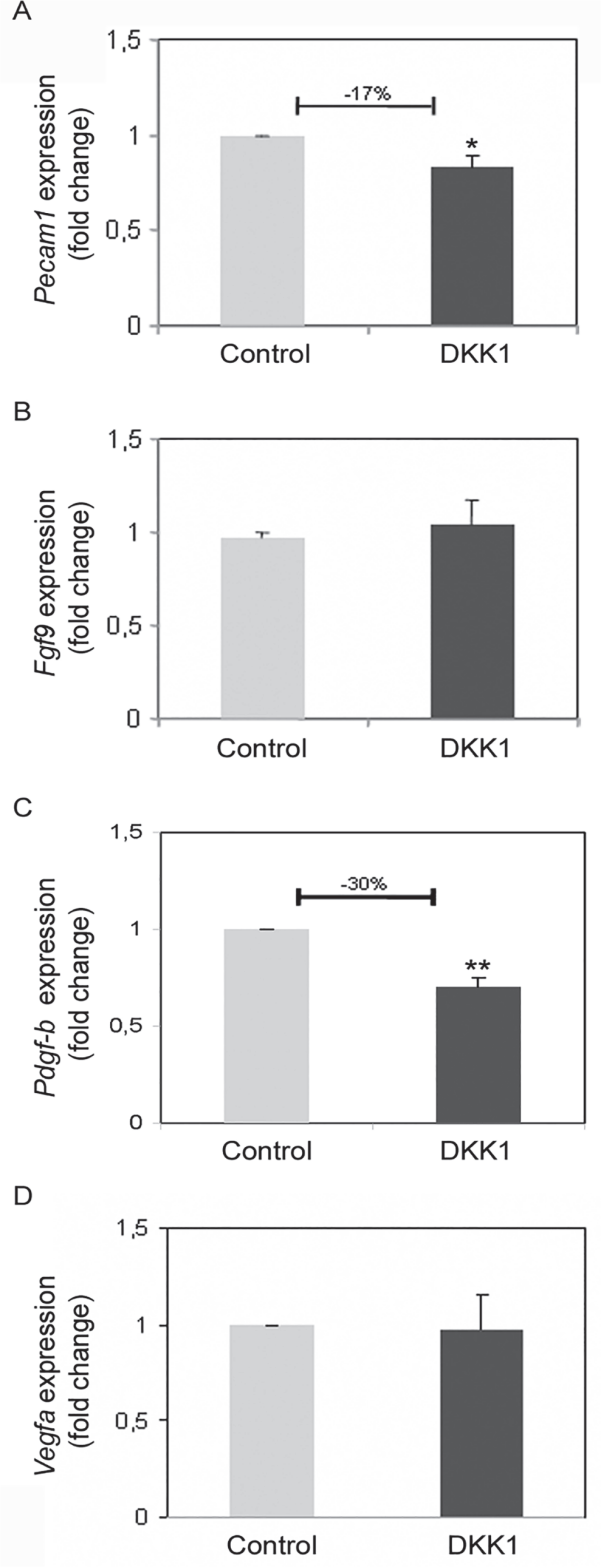
qPCR of cultured 12.5 dpc male UGRs. Real-time PCR analysis of *Pecam1* (A), *Fgf9* (B), *Pdgf-b* (C), and *Vegfa* (D) in control (light gray columns) and DKK1 treated samples (dark gray columns); the number depicted above the graphs (when reported) means the fold change of transcript expression in treated samples vs. control samples. Error bars indicate s.e.m. (*P < 0.05; ** P < 0.01).

In order to better characterize the reported DKK1 induced impairment of testicular angiogenesis, we performed Real-time PCR experiments analysing, in our *ex vivo* organ cultures, the expression level of some genes known to be involved in testicular determination (*Fgf9*), cordonogenesis (*Fgf9*, *Pdgf-b*), and angiogenesis (*Pecam1*, *Vegfa; Pdgf-b)*. Interestingly, we found that DKK1 is able to slightly, but significantly, decrease the expression level of the endothelial marker *Pecam1* (Fig 8A). Considering that *Pecam1* is a marker of both endothelial and germ cells, it is conceivable that the slight decrease in *Pecam1* expression might concern only testicular endothelial cell lineage: these cells are less represented compared to germ cells in the developing testis, at least at the age in which the organ culture experiments were performed.

Intriguingly, DKK1 administration was also able to perturb the expression level of the *Pdgf-b* chain (Fig 8C) without affecting *Fgf9* (Fig 8B) and *Vegfa* (Fig 8D) gene expression indicating that the alteration of WNT/RSPO signaling pathway might impair testicular angiogenesis acting on *Pdgf-b* availability.

### RSPO1 promotes the endothelial cell migration toward the developing testis

**Fig 9:**
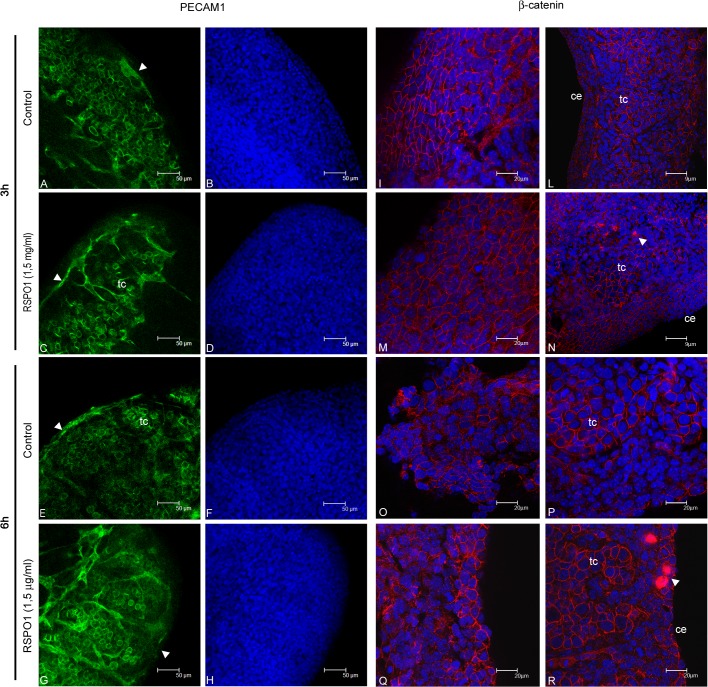
PECAM1 and β-catenin distribution pattern in 3 and 6 hours cultured 12.5 dpc male UGRs. Confocal microscopy analysis of PECAM1 distribution, observed by whole mount immunofluorescence in 12.5 dpc male gonads cultured for 3 (A-D) and 6 (E-H) hours in medium alone (A and E), and in the presence of RSPO1 (C and G). The corresponding TO-PRO3 staining (nuclei) are reported in B,D,F, and H. Arrowheads indicate the coelomic vessel. Confocal microscopy analysis of β-catenin distribution, observed by whole mount immunofluorescence in 12.5 dpc male gonads cultured for 3 (I-N) and 6 (O-R) hours in medium alone (I and L, O and P), and in the presence of RSPO1 (M and N, Q and R). All the images are merged with the respective TO-PRO3 staining. I,M,O, and Q show representative optical sections withdrawn at the organ surface while L, N, P, and R show representative optical sections withdrawn in the inner part of the same organs. ce: coelomic epithelium; tc: testicular cords. White arrowheads indicate β-catenin nuclear staining.

These results indicate that DKK1 is an inhibitor of normal testicular angiogenesis. We suggest that this may be the reason why DKK1 protein is physiologically never available on the testicular coelomic surface, since this area is an organizer of testis vasculature. Conversely, as reported, RSPO1 is clearly detectable in this region, which implies that this factor may be involved in the promotion of testicular angiogenesis. To test this hypothesis, we cultured 12.5 dpc male UGRs in the presence or in absence of 1,5 μg/ml RSPO1 for 3 and 6 hours. Samples were then fixed and processed to detect PECAM1 positive cell distribution pattern. Interestingly, we observed a well-defined internal vascularisation in RSPO1 treated testes, compared to the contra-lateral controls at all sampling times analysed (Fig 9A–9H).

The same culture conditions used to assess RSPO1-mediated endothelial cell migration, were also used to study RSPO1-mediated β-catenin activation. Interestingly, consistent with the results obtained on freshly isolated testes (Fig 3), we found that β-catenin nuclear translocation was also a relatively rare event in our *ex vivo* organ cultures. However, it is interesting to note that β-catenin nuclear patterning was observable only in RSPO1 treated samples and appeared to be restricted to very few cells of the interstitial compartment or the coelomic domain (Fig 9N and 9R). Noteworthy, we observed that, in the testicular cords, β-catenin never translocates in the nucleus both in the control and in the treated samples and regardless the culture time interval. Moreover, the tubular β-catenin patterning did not change after RSPO1 administration at any culture time interval analysed and was comparable to that reported in the freshly isolated foetal testes (Figs 3 and 9L, 9N, 9P and 9R). Interestingly, we observed that, after three hours of culture time, and in some portions of the coelomic surface, the β-catenin distribution pattern appeared slightly modified in the RSPO1 treated samples. In particular, β-catenin showed distribution both in the cortical part and in the inner part of the cytoplasm of these cells, whereas in the control samples, the localization of this protein was restricted to the cortical cytoplasm and was significantly sharper and clearly defined (Fig 9I and 9M). It is interesting to highlight that, after six hours of culture time, both the control and the RSPO1 treated samples showed, at the coelomic surface, the previously mentioned partial cytoplasmic delocalization of β-catenin, indicating that, this cytoplamic diffusion, is triggered by the signal molecules physiologically available in the testicular microenvironment (Fig 9O and 9Q).All together, these results confirm the previously described LGR4 distribution pattern (Fig 5), indicating that, in the foetal testis, the RSPO1 target cells are localized in the interstitial compartment and in coelomic domain.

## Discussion

Formation of the testis-specific vasculature is one of the earliest events that occurs to establish testis embryonic development. This process has been extensively studied by different groups [1;4–6]. However, the molecular cues outlining this morphogenetic event are still not completely understood. In mammals, gonad vascularisation is a sexually dimorphic process and the study of embryonic testicular micro-environmental signals, responsible for this phenomenon, could help define the diverging sex-specific pathways activated during the morphogenesis of male and female gonads. The research in this field poses a challenge from several perspectives: firstly, it will improve our understanding of the molecular alterations that outline intersex disorders; secondly, it will shed much light on our knowledge of the molecular underpinnings of tumour vascularisation, as stated by Coveney and colleagues in 2008 who highlighted important similarities between mechanisms of foetal testis and tumour vascular pattern setups.

Prenatal testis provides an excellent model for the study of vascularisation and several studies have demonstrated that, it is possible to analyse this phenomenon by *ex vivo* organ culture, even in the absence of blood circulation; this experimental model allows endothelial cells to migrate reflecting the normal *in vivo* vascular pattern [1;5;35]. Furthermore, it has been demonstrated, using *ex vivo* organ cultures, that testicular vasculogenesis has originated from the disassembly of mesonephric vessels. Mesonephric endothelial cells are activated by testis specific signals and migrate through the gonad towards the coelomic epithelium [4].

We know that RSPOs play important roles in various developmental processes such as myogenesis, mouse placental development, limb bud differentiation, neural tube morphogenesis [9;20;36–40] and that these roles are conserved among vertebrates. In particular, embryonic angiogenesis appears to be regulated by RSPOs: RSPO3 in Xenopus and mouse [39] as well as RSPO1 in zebrafish, are involved in this process [41]. Specifically, both these RSPOs do not appear to be fundamental in primary vessel vasculogenesis, but have been shown to play an important role in the subsequent angiogenesis. In addition, RSPO1 is used as a pro-angiongenic factor of the skin [42].

Recently, RSPO1 has proved to be a key ovary-determining factor [22–27] and may cooperate with WNT4 which has been shown to be a key regulator of ovary morphogenesis even before RSPO1 [29;43–45]. Many studies reveal that RSPO1 or WNT4 pathways are not involved [46] or are barely involved [23;47;48] in testis pre-natal development. However, it is worth highlighting that WNT signals present a sex-specific expression pattern in the developing gonads, being WNT4, WNT5a, WNT6 and WNT9 expressed in the ovary and WNT1, WNT3, and WNT7a expressed in the testis [46;49–53]. This observation indicates that the regulation of these molecular cues seems to be important not only for female, but also for male gonad differentiation. However, it is worth noting that the overlapping expression of multiple *Wnt* genes suggests that these molecules could act redundantly and/or synergistically: which suggest that the loss of expression of one of these genes may be vicariated by other similar cues. This observation could explain the lack of phenotype during embryonic morphogenesis when just one of these cues is knocked out and indicates how the high functional redundancy activated during gonad morphogenesis could complicate interpretation of gene functions.

We reported, in the introduction section, that RSPOs are unable to initiate WNT signaling, but they can potently enhance responses to low-dose WNT proteins. In this study we found that RSPO1 is always expressed in the developing testis from 11.5 to 18.5 dpc. Since WNT4 is down-regulated in the male soon after *Sry* expression, it is reasonable to suppose that this molecular cue could enhance the testis-specific WNT signals located in the testicular microenvironment. To the best of our knowledge, all these signals activate the common β-catenin signaling pathway and, it is likely that the biological effects triggered by β-catenin in male and female gonad are different due to the sex-specific cellular and molecular microenvironment in which this pathway is embedded.

As mentioned previously, this study started from our western blot analysis observations, which have revealed that RSPO1 protein was clearly detectable in different phases of prenatal testis development. Literature data show that RSPO1 is an ovary determinant factor, therefore we hypothesized that, in the developing testis, the availability of RSPO1 would be actively counteracted by the antagonist DKK1 or by the lack of the intracellular signal transducer β-catenin. It has been demonstrated by Liu and colleagues in 2009, that testicular morphogenesis develops normally in mice lacking the expression of β-catenin in SF1 male gonadal cells (Sertoli and Leydig cells). Thus, to investigate the potential role of RSPO1 in testicular morphogenesis, we studied the distribution pattern of RSPO1/DKK1/β-catenin machinery during the entire period of testicular pre-natal development. Interestingly and surprisingly, we found that, although DKK1 is always clearly detectable in the developing testis, its distribution pattern only partially overlaps RSPO1 localization in the testicular districts. More precisely, DKK1 is present in the entire parenchyma of male genital ridge at 11.5 dpc while RSPO1 is detectable only on its coelomic portion. The day after, at 12.5 dpc, we observed that DKK1 became restricted to the developing testicular cords whereas RSPO1 was clearly detectable on the coelomic epithelium and in the cranial gonad/mesonephros interface. From 13.5 dpc to 18.5 dpc both molecules appear clearly detectable in the testicular cords but only RSPO1, and not DKK1, is present in the developing coelomic region. Interestingly, β-catenin appears to be restricted to the areas in which RSPO1 is present namely on the coelomic surface and the testicular cords. We therefore hypothesized that RSPO1 pathway is active in the testicular ceolomic surface and is not counteracted by DKK1 in this area.

Since testicular coelomic epithelium is an important organizer of testicular angiogenesis, we studied the effect of RSPO1 and DKK1 on 12.5 dpc testis organ cultures and observed that DKK1 has an inhibitory effect on endothelial cell migration towards the testicular coelomic domain, whereas RSPO1 promotes testicular angiogenesis and rescues DKK1 inhibition of endothelial cell migration. Consistent with these data, Real-time PCR analyses allowed us to observe that both *Pecam1* and *Pdgf-b* chain expression level are depressed after DKK1 administration. It is well known that PDGF and PDGFR-alpha are involved in testicular cord formation, mesonephric cell migration, and testis specific vascular patterning [6;54;55]. Moreover, in a different biological model, it has been reported that WNT/β-catenin signaling modulates angiogenesis promoting *Pdgf-b* expression [56]. All together, these observations allowed us to hypothesize that WNT/RSPO signaling promotes, in the developing testis, *Pdgf-b* expression and that this phenomenon is in turn responsible for the testis specific angiogenesis.

Intriguingly, in our experimental conditions, DKK1 is not able to modulate *Vegfa* gene expression: this is absolutely compatible with our data, since we are not reporting a complete abrogation of testicular angiogenesis but a consistent reduced internal vascular branching mediated by DKK1.

We evaluated also *Fgf9* expression level, since it is a well known marker of testis determination [57]. Moreover, it has been reported that the lack of *Fgf9* during male development, results in the up-regulation of *Wnt4* and conversely, the loss of *Wnt4* is sufficient to up-regulate *Fgf9* expression [7;58]. As reported, *Fgf9* expression level does not change after DKK1 administration. This result indicates that DKK1 administration does not act against WNT4 signaling, at least at the developmental age of our *ex vivo* organ cultures, and, potentially, it can counteract the testis specific WNT/RSPO signaling pathways. We can conclude that DKK1 administration in 12.5 dpc male gonads does not affect testicular determination.

The results of the organ culture experiments were in line with the reported distribution pattern of LGR4 (RSPO1 receptor) and Kremen1 (DKK1 receptor): both these molecules are mainly detectable on the coelomic surface of the developing testes. Moreover, we found that some testicular endothelial cells express LGR4 receptor. Therefore, it is reasonable to suggest that testicular endothelial cells could, at least partially, respond directly to RSPO1.

It has been reported that β-catenin is dispensable for male gonad embryonic development using a conditional knockout model in which β-catenin was ablated in SF1 expressing somatic cells [46]. In the light of our data, however, the results of Liu and colleagues take on a different meaning: our immunofluorescence experiments clearly demonstrate that SF1 expressing cells (Sertoli cells and Leydig cells) do not appear to be the target of RSPO1/DKK1 signaling during testicular morphogenesis. As previously stated, in fact, LGR4 and Kremen1 are not detectable on these cell lineages. Thus, the lack of phenotype reported in the β-catenin conditional knockout model could depend on the cell types in which the β-catenin was knocked out and not on the dispensability of β-catenin in male gonad development.

Interestingly, our results support the data reported by Gore and colleagues in 2011. They observed that zebrafish embryos lacking *rspo1* form primary vessels by normal vasculogenesis, but are defective in subsequent angiogenesis and that endothelial cell-autonomous inhibition of canonical WNT signaling also blocks angiogenesis *in vivo* [41]. These data indicate the highly conserved role of RSPO1 in the regulation of embryonic vascular branching. Moreover, the identification of RSPO1 as a secreted angiogenic factor for the embryonic testis may provide novel opportunities for pharmaceutical intervention in angiogenic and antiangiogenic therapies [59;60].

In conclusion, our results expand our knowledge of RSPO1 physiological roles, shedding light on the understanding of the molecular control of testis morphogenesis: we demonstrate, in fact, for the first time, that RSPO1 is present in all the phases of testicular morphogenesis and that it has a role in the regulation of the testicular vascular pattern determination, probably acting via *Pdgf-b* gene expression. These data strongly indicate the complexity and redundancy of the prenatal testis microenvironment with a potential to respond robustly to genetic or epigenetic alterations. Since testis angiogenesis seems to have some similarities with tumour angiogenesis, the role of RSPO1, in tumour progression and vascularisation, could represent a common mechanism used in physiological and pathological angiogenesis. From this viewpoint, the molecular pathway crosstalk activated by RSPO1 in the testicular context deserves further investigations.

## Supporting Information

S1 Fig β-catenin immunofluorescence negative controls. Confocal microscopy analysis of the TRITC anti-mouse antibody background (β-catenin immunofluorescence negative controls) observed in 12.5 (A), 15.5 (C), and 18.5 dpc (E) male urogenital ridges. The corresponding TO-PRO3 staining (nuclei) are reported in B, D, and F.ts: testis; tc: testicular cords
(TIF)

S2 Fig PECAM1 distribution pattern in 12.5, 13.5 and 15.5 dpc male gonads. Confocal microscopy analysis of PECAM1 distribution, observed by whole mount immunofluorescence in 12.5, 13.5 and 15.5 dpc male UGRs. PECAM1 (FITC signal) in 12.5 dpc male UGRs, observed at different magnifications, is reported in A and C; images B and D show the corresponding TO-PRO3 staining (nuclei). Images E and G show PECAM1 (FITC signal) in 13.5 and 15.5 dpc male UGRs respectively; images F and H show the corresponding TO-PRO3 staining (nuclei). ms: mesonephros; ts: testis; white arrowheads indicate celomic vessel.
(TIF)

## References

[1] Brennan J, Karl J, Capel B. Divergent vascular mechanisms downstream of Sry establish the arterial system in the XY gonad. Dev Biol. 2002; 244:418–428. 11944948 10.1006/dbio.2002.0578

[2] Byskov AG. Differentiation of mammalian embryonic gonad. Physiol Rev. 1986; 66:71–117. 3511481 10.1152/physrev.1986.66.1.71

[3] Nagamine CM, Carlisle C. The dominant white spotting oncogene allele Kit(W-42J) exacerbates XY(DOM) sex reversal. Development. 1996; 122:3597–3605. 8951075 10.1242/dev.122.11.3597

[4] Coveney D, Cool J, Oliver T, Capel B. Four-dimensional analysis of vascularization during primary development of an organ, the gonad. Proc Natl Acad Sci U S A. 2008; 105:7212–7217. 10.1073/pnas.0707674105 18480267 PMC2438229

[5] Combes AN, Wilhelm D, Davidson T, Dejana E, Harley V, Sinclair A, et al Endothelial cell migration directs testis cord formation. Dev Biol. 2009; 326:112–120. 10.1016/j.ydbio.2008.10.040 19041858

[6] Cool J, DeFalco TJ, Capel B. Vascular-mesenchymal cross-talk through Vegf and Pdgf drives organ patterning. Proc Natl Acad Sci U S A. 2011; 108:167–172. 10.1073/pnas.1010299108 21173261 PMC3017142

[7] Jameson SA, Lin YT, Capel B. Testis development requires the repression of Wnt4 by Fgf signaling. Dev Biol. 2012; 370:24–32. 10.1016/j.ydbio.2012.06.009 22705479 PMC3634333

[8] Chen JZ, Wang S, Tang R, Yang QS, Zhao E, Chao Y, et al Cloning and identification of a cDNA that encodes a novel human protein with thrombospondin type I repeat domain, hPWTSR. Mol Biol Rep. 2002; 29:287–292. 12463421 10.1023/a:1020479301379

[9] Kamata T, Katsube K, Michikawa M, Yamada M, Takada S, Mizusawa H. R-spondin, a novel gene with thrombospondin type 1 domain, was expressed in the dorsal neural tube and affected in Wnts mutants. Biochim Biophys Acta. 2004; 1676:51–62. 14732490 10.1016/j.bbaexp.2003.10.009

[10] Kazanskaya O, Glinka A, del BB I, Stannek P, Niehrs C, Wu W. R-Spondin2 is a secreted activator of Wnt/beta-catenin signaling and is required for Xenopus myogenesis. Dev Cell. 2004; 7:525–534. 15469841 10.1016/j.devcel.2004.07.019

[11] Kim KA, Kakitani M, Zhao J, Oshima T, Tang T, Binnerts M, et al Mitogenic influence of human R-spondin1 on the intestinal epithelium. Science. 2005; 309:1256–1259. 16109882 10.1126/science.1112521

[12] Kim KA, Zhao J, Andarmani S, Kakitani M, Oshima T, Binnerts ME, et al R-Spondin proteins: a novel link to beta-catenin activation. Cell Cycle. 2006; 5:23–26. 16357527 10.4161/cc.5.1.2305

[13] Nam JS, Turcotte TJ, Yoon JK. Dynamic expression of R-spondin family genes in mouse development. Gene Expr Patterns. 2007; 7:306–312. 17035101 10.1016/j.modgep.2006.08.006

[14] de Lau WB, Snel B, Clevers HC. The R-spondin protein family. Genome Biol. 2012; 13:242 10.1186/gb-2012-13-3-242 22439850 PMC3439965

[15] Glinka A, Dolde C, Kirsch N, Huang YL, Kazanskaya O, Ingelfinger D, et al LGR4 and LGR5 are R-spondin receptors mediating Wnt/beta-catenin and Wnt/PCP signalling. EMBO Rep. 2011; 12:1055–1061. 10.1038/embor.2011.175 21909076 PMC3185347

[16] Binnerts ME, Kim KA, Bright JM, Patel SM, Tran K, Zhou M, et al R-Spondin1 regulates Wnt signaling by inhibiting internalization of LRP6. Proc Natl Acad Sci U S A. 2007; 104:14700–14705. 17804805 10.1073/pnas.0702305104 PMC1965484

[17] Nusse R. Wnt signaling in disease and in development. Cell Res. 2005; 15:28–32. 15686623 10.1038/sj.cr.7290260

[18] Boudin E, Fijalkowski I, Piters E, Van HW. The role of extracellular modulators of canonical Wnt signaling in bone metabolism and diseases. Semin Arthritis Rheum. 2013; 43:220–240. 10.1016/j.semarthrit.2013.01.004 23433961

[19] Niehrs C. Function and biological roles of the Dickkopf family of Wnt modulators. Oncogene. 2006; 25:7469–7481. 17143291 10.1038/sj.onc.1210054

[20] Yoon JK, Lee JS. Cellular signaling and biological functions of R-spondins. Cell Signal. 2012; 24:369–377. 10.1016/j.cellsig.2011.09.023 21982879 PMC3237830

[21] Biason-Lauber A. Control of sex development. Best Pract Res Clin Endocrinol Metab. 2010; 24:163–186. 10.1016/j.beem.2009.12.002 20541146

[22] Chassot AA, Ranc F, Gregoire EP, Roepers-Gajadien HL, Taketo MM, Camerino G, et al Activation of beta-catenin signaling by Rspo1 controls differentiation of the mammalian ovary. Hum Mol Genet. 2008; 17:1264–1277. 10.1093/hmg/ddn016 18250098

[23] Chassot AA, Gregoire EP, Lavery R, Taketo MM, de Rooij DG, Adams IR, et al RSPO1/beta-catenin signaling pathway regulates oogonia differentiation and entry into meiosis in the mouse fetal ovary. PLoS One. 2011; 6:e25641 10.1371/journal.pone.0025641 21991325 PMC3185015

[24] Chassot AA, Bradford ST, Auguste A, Gregoire EP, Pailhoux E, de Rooij DG, et al WNT4 and RSPO1 together are required for cell proliferation in the early mouse gonad. Development. 2012; 139:4461–4472. 10.1242/dev.078972 23095882

[25] Parma P, Radi O, Vidal V, Chaboissier MC, Dellambra E, Valentini S, et al R-spondin1 is essential in sex determination, skin differentiation and malignancy. Nat Genet. 2006; 38:1304–1309. 17041600 10.1038/ng1907

[26] Tomaselli S, Megiorni F, De Bernardo C, Felici A, Marrocco G, Maggiulli G, et al Syndromic true hermaphroditism due to an R-spondin1 (RSPO1) homozygous mutation. Hum Mutat. 2008; 29:220–226. 18085567 10.1002/humu.20665

[27] Tomaselli S, Megiorni F, Lin L, Mazzilli MC, Gerrelli D, Majore S, et al Human RSPO1/R-spondin1 is expressed during early ovary development and augments beta-catenin signaling. PLoS One. 2011; 6:e16366 10.1371/journal.pone.0016366 21297984 PMC3030573

[28] Tomizuka K, Horikoshi K, Kitada R, Sugawara Y, Iba Y, Kojima A, et al R-spondin1 plays an essential role in ovarian development through positively regulating Wnt-4 signaling. Hum Mol Genet. 2008; 17:1278–1291. 10.1093/hmg/ddn036 18250097

[29] Vainio S, Heikkila M, Kispert A, Chin N, McMahon AP. Female development in mammals is regulated by Wnt-4 signalling. Nature. 1999; 397:405–409. 9989404 10.1038/17068

[30] Pesce M, De Felici M. Purification of mouse primordial germ cells by MiniMACS magnetic separation system. Dev Biol. 1995; 170:722–725. 7649397 10.1006/dbio.1995.1250

[31] Ciccarone F, Klinger FG, Catizone A, Calabrese R, Zampieri M, Bacalini MG, et al Poly(ADP-ribosyl)ation acts in the DNA demethylation of mouse primordial germ cells also with DNA damage-independent roles. PLoS One. 2012; 7:e46927 10.1371/journal.pone.0046927 23071665 PMC3465317

[32] Fedi P, Bafico A, Nieto SA, Burgess WH, Miki T, Bottaro DP, et al Isolation and biochemical characterization of the human Dkk-1 homologue, a novel inhibitor of mammalian Wnt signaling. J Biol Chem. 1999; 274:19465–19472. 10383463 10.1074/jbc.274.27.19465

[33] Livak KJ, Schmittgen TD. Analysis of relative gene expression data using real-time quantitative PCR and the 2(-Delta Delta C(T)) Method. Methods. 2001; 25:402–408. 11846609 10.1006/meth.2001.1262

[34] Combes AN, Bowles J, Feng CW, Chiu HS, Khoo PL, Jackson A, et al Expression and functional analysis of Dkk1 during early gonadal development. Sex Dev. 2011; 5:124–130. 10.1159/000327709 21654186 PMC3121542

[35] Tilmann C, Capel B. Mesonephric cell migration induces testis cord formation and Sertoli cell differentiation in the mammalian gonad. Development. 1999; 126:2883–2890. 10357932 10.1242/dev.126.13.2883

[36] Aoki M, Mieda M, Ikeda T, Hamada Y, Nakamura H, Okamoto H. R-spondin3 is required for mouse placental development. Dev Biol. 2007; 301:218–226. 16963017 10.1016/j.ydbio.2006.08.018

[37] Bell SM, Schreiner CM, Wert SE, Mucenski ML, Scott WJ, Whitsett JA. R-spondin 2 is required for normal laryngeal-tracheal, lung and limb morphogenesis. Development. 2008; 135:1049–1058. 10.1242/dev.013359 18256198

[38] Han XH, Jin YR, Seto M, Yoon JK. A WNT/beta-catenin signaling activator, R-spondin, plays positive regulatory roles during skeletal myogenesis. J Biol Chem. 2011; 286:10649–10659. 10.1074/jbc.M110.169391 21252233 PMC3060516

[39] Kazanskaya O, Ohkawara B, Heroult M, Wu W, Maltry N, Augustin HG, et al The Wnt signaling regulator R-spondin 3 promotes angioblast and vascular development. Development. 2008; 135:3655–3664. 10.1242/dev.027284 18842812

[40] Nam JS, Park E, Turcotte TJ, Palencia S, Zhan X, Lee J, et al Mouse R-spondin2 is required for apical ectodermal ridge maintenance in the hindlimb. Dev Biol. 2007; 311:124–135. 17904116 10.1016/j.ydbio.2007.08.023 PMC2692258

[41] Gore AV, Swift MR, Cha YR, Lo B, McKinney MC, Li W, et al Rspo1/Wnt signaling promotes angiogenesis via Vegfc/Vegfr3. Development. 2011; 138:4875–4886. 10.1242/dev.068460 22007135 PMC3201658

[42] Kuppan P, Vasanthan KS, Sundaramurthi D, Krishnan UM, Sethuraman S. Development of poly(3-hydroxybutyrate-co-3-hydroxyvalerate) fibers for skin tissue engineering: effects of topography, mechanical, and chemical stimuli. Biomacromolecules. 2011; 12:3156–3165. 10.1021/bm200618w 21800891

[43] Biason-Lauber A, Konrad D, Navratil F, Schoenle EJ. A WNT4 mutation associated with Mullerian-duct regression and virilization in a 46,XX woman. N Engl J Med. 2004; 351:792–798. 15317892 10.1056/NEJMoa040533

[44] Biason-Lauber A, De Filippo G, Konrad D, Scarano G, Nazzaro A, Schoenle EJ. WNT4 deficiency—a clinical phenotype distinct from the classic Mayer-Rokitansky-Kuster-Hauser syndrome: a case report. Hum Reprod. 2007; 22:224–229. 16959810 10.1093/humrep/del360

[45] Biason-Lauber A, Konrad D. WNT4 and sex development. Sex Dev. 2008; 2:210–218. 10.1159/000152037 18987495

[46] Liu CF, Bingham N, Parker K, Yao HH. Sex-specific roles of beta-catenin in mouse gonadal development. Hum Mol Genet. 2009; 18:405–417. 10.1093/hmg/ddn362 18981061 PMC2638797

[47] Jeays-Ward K, Hoyle C, Brennan J, Dandonneau M, Alldus G, Capel B, et al Endothelial and steroidogenic cell migration are regulated by WNT4 in the developing mammalian gonad. Development. 2003; 130:3663–3670. 12835383 10.1242/dev.00591

[48] Jeays-Ward K, Dandonneau M, Swain A. Wnt4 is required for proper male as well as female sexual development. Dev Biol. 2004; 276:431–440. 15581876 10.1016/j.ydbio.2004.08.049

[49] Bouma GJ, Hart GT, Washburn LL, Recknagel AK, Eicher EM. Using real time RT-PCR analysis to determine multiple gene expression patterns during XX and XY mouse fetal gonad development. Gene Expr Patterns. 2004; 5:141–149. 15533830 10.1016/j.modgep.2004.05.001

[50] Cederroth CR, Pitetti JL, Papaioannou MD, Nef S. Genetic programs that regulate testicular and ovarian development. Mol Cell Endocrinol. 2007; 265–266:3–9. 10.1016/j.mce.2006.12.029 17208359

[51] Erickson RP, Lai LW, Grimes J. Creating a conditional mutation of Wnt-1 by antisense transgenesis provides evidence that Wnt-1 is not essential for spermatogenesis. Dev Genet. 1993; 14:274–281. 8222343 10.1002/dvg.1020140405

[52] Katoh M. Molecular cloning and characterization of human WNT3. Int J Oncol. 2001; 19:977–982. 11604997 10.3892/ijo.19.5.977

[53] Kirikoshi H, Katoh M. Expression of WNT7A in human normal tissues and cancer, and regulation of WNT7A and WNT7B in human cancer. Int J Oncol. 2002; 21:895–900. 12239632

[54] Brennan J, Tilmann C, Capel B. Pdgfr-alpha mediates testis cord organization and fetal Leydig cell development in the XY gonad. Genes Dev. 2003; 17:800–810. 12651897 10.1101/gad.1052503 PMC196020

[55] Ricci G, Catizone A, Galdieri M. Embryonic mouse testis development: role of platelet derived growth factor (PDGF-BB). J Cell Physiol. 2004; 200:458–467. 15254974 10.1002/jcp.20035

[56] Reis M, Czupalla CJ, Ziegler N, Devraj K, Zinke J, Seidel S, et al Endothelial Wnt/beta-catenin signaling inhibits glioma angiogenesis and normalizes tumor blood vessels by inducing PDGF-B expression. J Exp Med. 2012; 209:1611–1627. 10.1084/jem.20111580 22908324 PMC3428944

[57] Colvin JS, Green RP, Schmahl J, Capel B, Ornitz DM (2001) Male-to-female sex reversal in mice lacking fibroblast growth factor 9. Cell 104:875–889. 11290325 10.1016/s0092-8674(01)00284-7

[58] Kim Y, Kobayashi A, Sekido R, DiNapoli L, Brennan J, Chaboissier MC, et al Fgf9 and Wnt4 act as antagonistic signals to regulate mammalian sex determination. PLoS Biol. 2006; 4:e187 16700629 10.1371/journal.pbio.0040187 PMC1463023

[59] Dor Y, Djonov V, Keshet E. Induction of vascular networks in adult organs: implications to proangiogenic therapy. Ann N Y Acad Sci. 2003; 995:208–216. 12814953 10.1111/j.1749-6632.2003.tb03224.x

[60] Ferrara N, Alitalo K. Clinical applications of angiogenic growth factors and their inhibitors. Nat Med. 1999; 5:1359–1364. 10581076 10.1038/70928

