# Supplementary figures and images for: R-Spondin 1/Dickkopf-1/Beta-Catenin Machinery Is Involved in Testicular Embryonic Angiogenesis
Source: PLoS One. 2015 Apr 24;10(4):e0124213. doi: 10.1371/journal.pone.0124213 (PMC4409372; doi:10.1371/journal.pone.0124213)

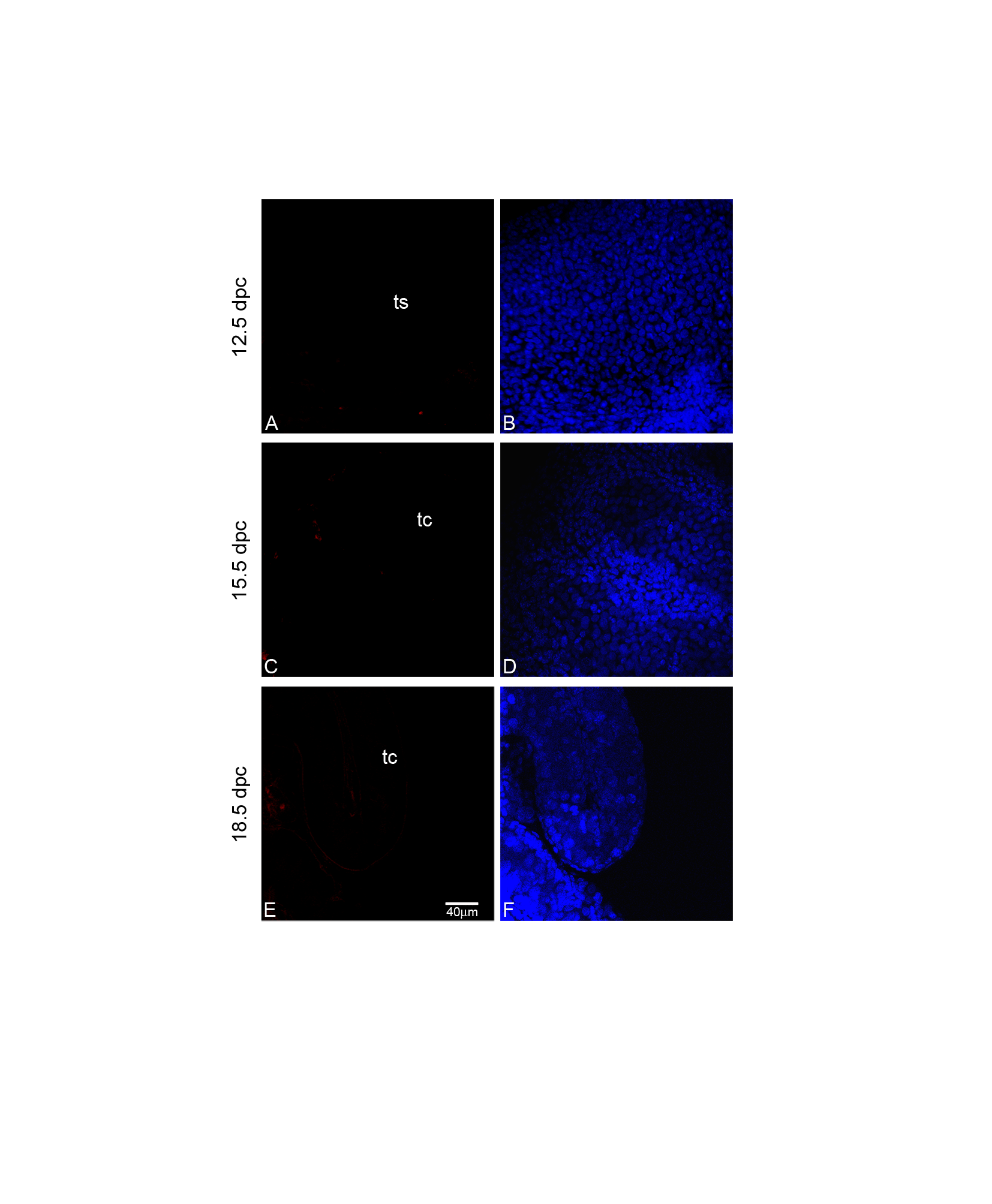

Supplement: S1 Fig — Confocal microscopy analysis of the TRITC anti-mouse antibody background (β-catenin immunofluorescence negative controls) observed in 12.5 (A), 15.5 (C), and 18.5 dpc (E) male urogenital ridges. The corresponding TO-PRO3 staining (nuclei) are reported in B, D, and F.ts: testis; tc: testicular cords (TIF) [file pone.0124213.s001.tif]

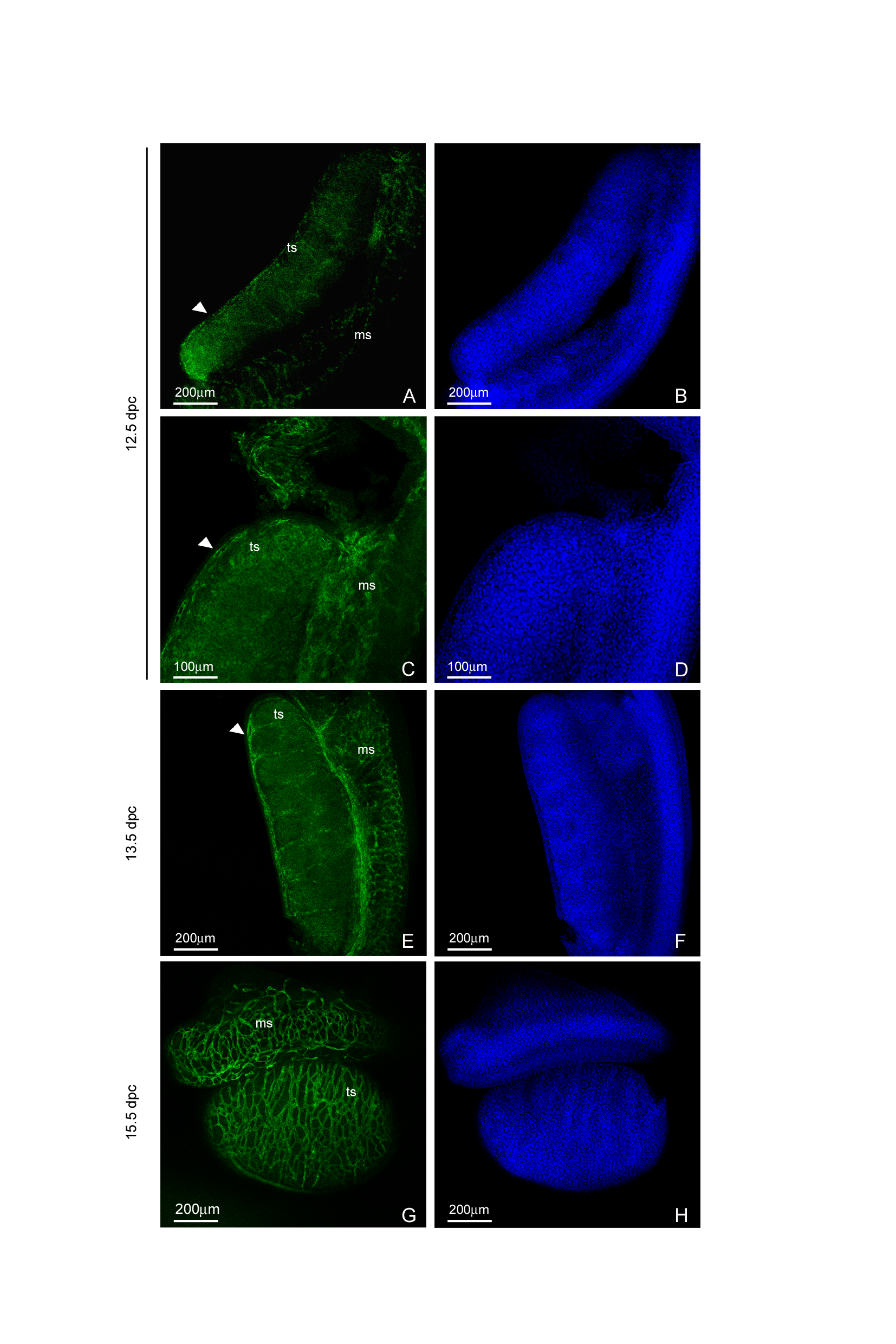

Supplement: S2 Fig — Confocal microscopy analysis of PECAM1 distribution, observed by whole mount immunofluorescence in 12.5, 13.5 and 15.5 dpc male UGRs. PECAM1 (FITC signal) in 12.5 dpc male UGRs, observed at different magnifications, is reported in A and C; images B and D show the corresponding TO-PRO3 staining (nuclei). Images E and G show PECAM1 (FITC signal) in 13.5 and 15.5 dpc male UGRs respectively; images F and H show the corresponding TO-PRO3 staining (nuclei). ms: mesonephros; ts: testis; white arrowheads indicate celomic vessel. (TIF) [file pone.0124213.s002.tif]
